# Improving the Method of Short-term Forecasting of Electric Load in Distribution Networks using Wavelet transform combined with Ridgelet Neural Network Optimized by Self-adapted Kho-Kho Optimization Algorithm

**DOI:** 10.1016/j.heliyon.2024.e28381

**Published:** 2024-03-26

**Authors:** Yaoying Wang, Shudong Sun, Gholamreza Fathi, Mahdiyeh Eslami

**Affiliations:** a School of Mechanical Engineering, Northwestern Polytechnical University, Xi'an, 710072, Shaanxi, China; b Department of Electrical Engineering, Power & Water University of Technology (PWUT), Tehran, Iran; c Department of Electrical Engineering, Kerman Branch, Islamic Azad University, Kerman, Iran; d College of Technical Engineering, The Islamic University, Najaf, Iraq

**Keywords:** Electric load forecasting, Ridgelet neural network, Wavelet transform, Self-adapted kho-kho algorithm, Optimization

## Abstract

This paper proposes a new method for short-term electric load forecasting using a Ridgelet Neural Network (RNN) combined with a wavelet transform and optimized by a Self-Adapted (SA) Kho-Kho algorithm (SAKhoKho). The aim of this method is to improve the accuracy and reliability of electric load forecasting, which is essential for the planning and operation of competitive electrical networks. The proposed method uses the Wavelet Transform (WT) to decompose the load data into different frequency components and applies the RNN to each component separately. The RNN is, then, optimized by the SAKhoKho algorithm, which is an improved version of the KhoKho algorithm that can adapt the search parameters dynamically. The proposed method is trained and tested on the Zone Preliminary Billing Data from the PJM regulatory area, which is updated every two weeks based on the Intercontinental Exchange (ICE) figures. The proposed method is compared with six other cutting-edge methods from the literature, including SVM/SA, hybrid, ARIMA, MLP/PSO, CNN, and RNN/KhoKho/WT. The results show that the proposed method achieves the lowest Mean Absolute Error (MAE) of 7.7704 and Root Mean Square Error (RMSE) of 17.4132 among all the methods, indicating its superior performance. The proposed method can capture the temporal dependencies in the load data and optimize the RNN's weights to minimize the error function. The proposed method is a promising technique for electric load forecasting, as it can provide accurate and reliable predictions for the next hour based on the previous 24 h of data.

## 1 Introduction

In recent years, industrial operations and social, financial, and agricultural activities are dependent on electricity, which makes it one of the most fundamental needs of human societies. Because the power supply controls various activities, it has high reliability and consistency [1].

In today's expanding and developing societies, the power sector plays a crucial and fundamental role. Production planning should respond to the fluctuating demand for electrical energy. This is because power industry projects take time and require significant investments, and with current technology, it is still impossible to store such a valuable resource on a large scale.

Estimating electric load consumption is a key element in designing and implementing energy procedures, as it enables efficient resource allocation for the growth and effective operation of the power supply network. The accuracy of load consumption prediction is crucial for developing the infrastructure of the electricity production and distribution system. If the predicted load is less than the actual load, the reliability factor and quality of service may decline, and in some cases, forced shutdowns may result.

Accurate load forecasting is crucial for efficient management of the power supply network, enabling utilities to make informed decisions about resource allocation, maintenance, and expansion, leading to better service and reduced costs for consumers. However, overestimating future load can result in significant investment losses [2]. Accurate load prediction has been found to be essential for planning and operating decisions in the energy generation and distribution system, resulting in increased customer satisfaction, energy savings, and service quality [3]. Load forecasting is critical for power companies, as variations in supply and demand, weather changes, and peak-hour energy price increases can impact the management of electric energy generation and distribution [4].

Therefore, low error rates in load forecasting are crucial for the future design and scheduling of an energy system [5]. The accuracy of load forecasting directly impacts the cost of production and distribution and the frequency of power outages. Efficient management of electric energy generation and distribution requires careful planning, operations, and investment maximization, making load forecasting of particular importance [6].

Neural networks, which simulate the structure and function of the human brain by the use of a computer system, are a new technique for estimating electrical load and energy, primarily for predicting very short-term and short-term loads [7]. Artificial neural networks are a popular model that has been extensively studied in the field of load prediction using AI methods [8]. However, the accuracy of surface artificial neural networks can be unsatisfactory in complex electric charge behavior situations [9], where deep learning techniques can be applied [10].

Deep belief, recurrent, and convolutional neural networks are among the deep learning techniques that have gained attention for their potential to improve prediction accuracy [11]. To this end, some research has been conducted on electric charge prediction using artificial intelligence techniques, for example Hafeez et al. [12] developed deep-learning-based load prediction optimized through heuristic algorithms in a smart network. Accurate load forecasting is crucial for decision-making about the power network and its operation, and there is a pressing need for accurate projecting models despite extensive research in this field. The proposed approach is a hybrid structure of feature choice modules, data pre-processing, optimizing, predicting, and training modules. The suggested pattern is verified by comparing its accuracy and convergence rate to those of four current prediction models, including Long Short-Term Memory (LSTM), Artificial Neural Network based on Mutual Information (MI-ANN), and bi-level. The outcomes demonstrate that the proposed Genetic Wind-Driven Optimizer (GWDO) improves the forecast precision and convergence value. The proposed model presents some advantages, including its integrated framework of data pre-processing and feature selection, deep learning, and optimization techniques, potentially improving forecasting accuracy. The Modified Mutual Information (MMI) used in the data pre-processing and feature selection module is an improved version of the mutual information technique, selecting more informative features from historical data. The Factored Conditional Restricted Boltzmann Machine (FCRBM) used in the training and forecasting module is a deep learning model that can capture complex patterns in electric load data, improving forecasting accuracy. The GWDO algorithm used in the optimization module can potentially optimize the adjustable parameters of the model more efficiently than other optimization algorithms. The proposed model's evaluation of historical hourly load data of three USA power grids adds to its generalizability. However, the article lacks detailed explanations of the MMI technique and the FCRBM model as well as training, making it difficult for readers to understand and replicate these techniques. Additionally, the comparison of the proposed model with only four recent forecasting models may not provide a comprehensive evaluation of the proposed model's performance.

Dai et al. [13] proposed a combined support vector machine-based load prediction model that included intelligent characteristic selection and variable optimization techniques. Support vector machines were widely used in power load prediction and were an excellent forecasting tool. The hybrid model suggested in this paper improved prediction accuracy by incorporating intelligent approaches for characteristic selection and parameter optimization. The study found that improving upon the Support Vector Machine (SVM) was considered to be effective, and the proposed technique outperformed other models in terms of stability, accuracy, and effectiveness that was demonstrated by comparing the forecasted outcomes of the proposed approach, the SVM before improvement, and three other prediction techniques.

Talaat et al. [14] utilized a Multilayer Feed-Forward Neural Network (MFFNN) with a regressive tactic and load prediction using Grasshopper optimization. To obtain highly accurate outcomes for load estimation, a hybrid method of MFFNN and Grasshopper Optimization Algorithm (GOA) were presented, known as the Mid-Term to Short-Term Load Forecasting (MT-STLF) technique. The study introduced a regression model that examined the relationships between the conditional parameter (load) and unconditional parameters that affect the load such as temperature. The regression model emphasized how temperature affected hourly load. The hybrid model's accuracy was satisfactory, with a deviation error ranging between −0.06 and 0.06. The proposed model had some advantages, including potentially improving the accuracy of load estimation.

Xie et al. [15] utilized Elman Neural Network (ENN) with Particle Swarm Optimization (PSO) to forecast short-term electricity load. The paper suggested combining ENN and PSO for short-term power load forecasting. First, the basics of ENN and PSO were introduced, followed by an analysis of how ENN parameters affected network performance. The particle swarm optimization algorithm was, then, used to find the best ENN learning rate. The effectiveness of the PSO-ENN approach was demonstrated by comparing it with General Regression Neural Network (GRNN), Novel Evolutionary NN (ENN), and conventional Back-Propagation NN (BPNN). The challenge of short-term electric charge prediction was utilized to test the effectiveness of the approach.

The advantages of the model included potentially improving the accuracy of short-term electricity load forecasting. The paper introduced the basics of ENN and PSO and analyzed how ENN parameters affected network's performance. The PSO was, then, used to find the best ENN learning rate. The effectiveness of the PSO-ENN was demonstrated by comparing it with GRNN, ENN, and conventional BPNN. The approach's effectiveness was tested on the challenge of short-term electric charge prediction. However, the article did not provide detailed explanations of the ENN and PSO techniques, making it difficult for readers to understand and replicate these techniques. Additionally, the comparison of the proposed approach with only three other forecasting models may not provide a comprehensive evaluation of the approach's performance.

Singh et al. [16] proposed a novel hybrid method that combined multi-objective optimization and neural networks for efficient load forecasting. They presented a new Multi-Objective Follow Leader optimizer (MOFTL) to achieve two goals simultaneously. The effectiveness of MOFTL was demonstrated by comparing the outcomes with three recently presented multi-objective optimizers, Multi-Objective Particle Swarm Optimizer (MOPSO), Multi-Objective Water Cycle Algorithm (MOWCA), and Non-Dominated Sorting GA (NSGA-II). The proposed combined model performed better than baseline models over two actual electricity data sets, England and the ERCOT areas. In comparison to other models, the proposed one exhibited some improvement in Mean Absolute Percentage Error (MAPE) values of 17.42%, 6.81%, 10.77%, and 59.69% for the England region and 4.20%, 4.16%, 1.14%, and 21.85% for the ERCOT region.

The proposed model had some advantages, including potentially improving the efficiency of load forecasting. The study introduced a new MOFTL that achieved two goals simultaneously and demonstrated its effectiveness by comparing the outcomes with three recently presented multi-objective optimizers. However, the article did not provide detailed explanations of the MOFTL optimizer and the neural network model used, making it difficult for readers to understand and replicate these techniques. Additionally, the comparison of the proposed approach with only 3 other multi-objective optimizers may not provide a comprehensive evaluation of the approach's performance.

Yin and Mao [17] introduced a novel fractional multi-variate grey Bernoulli model, denoted as MFGBM, which served as a means of predicting power load in the short term. The model incorporated fractional differential equation and fractional accumulation generation, thereby accounting for potential nonlinear relationships between power load and influencing factors. Furthermore, the grey wolf algorithm was enhanced to optimize various parameters in the model through a chaotic Tent map, inertia weights, a nonlinear function, and Lévy flight, thereby enhancing both local exploitation and global exploration capabilities. The proposed methodology was assessed using daytime and nighttime power loads in Australia, with electricity price, humidity, and temperature as influencing factors. Results indicated that MFGBM (q, r, N) was a highly effective approach for short-term power system prediction. Overall, this research provided a significant contribution to the field of power load prediction, presenting a new and effective avenue for addressing the challenges of accurate power load forecasting. The model offered some advantages for short-term power load prediction, including the incorporation of fractional differential equations and fractional accumulation generation to account for potential nonlinear relationships between power load and influencing factors. The enhanced GWO optimizes various parameters in the model, improving both local exploitation and global exploration capabilities. However, the article did not provide detailed explanations of the grey wolf algorithm and the MFGBM model, making it difficult for readers to understand and replicate these techniques. Additionally, the proposed approach was only evaluated using data from Australia, limiting the generalizability of the results to other regions.

This paper attempts to create a proper method for power consumption estimation techniques. The main information is separated through Discrete Wavelet Transform (DWT) transformation into components, depending on different scales or frequencies before entering the input layer of the Ridgelet Neural Network. Each of the components is, then, modified separately within the Ridgelet NN. The final forecasting is created by combining the independent units' time series projections. After that, the proposed strategy is applied to forecast an information set for energy demand. The proposed RNN/SA KhoKho/WT model offers a superior solution for load forecasting in comparison to traditional and machine learning methods. The use of wavelet transforms, Ridgelet Neural Network, and SA KhoKho algorithm provides a novel approach that captures temporal dependencies, nonlinear relationships and optimizes the model's weights and biases to improve accuracy. The study's contribution is in providing a more accurate and reliable method for short-term energy demand prediction, which can benefit power system operators in making informed decisions about energy generation and distribution. The proposed method's advantages over existing approaches are its ability to handle nonlinear relationships, its high accuracy, and its ability to capture temporal dependencies in the data.

## 2 Self-adapted Kho-Kho optimization algorithm

### 2.1 Mathematical modeling of chase policy

In this part, the mathematical simulation of runners and a pursuer on the playing field, as well as the techniques utilized by the pursuer to follow and touch a runner have been clarified.

#### 2.1.1 Identifying the sitting players’ sort and runner state

At the outset of a chase, it is beneficial to know the precise situation of the runner. In the represented optimizing method, the runner's place $\left({\vec{X}}_{r}\left(t\right)\right)$ belongs to the most suitable individual. At first, pursuers settle in a queue of blocks in a way that two sequential players in the pursuing set are located in front of each other. Accordingly, two sets of pursuers are shaped due to this facing. The players have been categorized into two sets based on a number. At this number, the player is located in the pursuing blocks' queue. Accordingly, the pursuers have been sorted based on the odd-numbered or the even-numbered state. The even-numbered pursuer set is specified as:

(1) $$P_{j}\left(j=2,4,6\ldots N\right)\subset P_{i},\left(i=1,2,3,\ldots N\right)$$

The odd-numbered pursuer set is also clarified as:

(2) $$P_{j}\left(j=1,3,5\ldots N-1\right)\subset P_{i},\left(i=1,2,3,\ldots N\right)$$

#### 2.1.2 Pursuer selection

Pursuer is a person who follows a runner. A player who confronts the existing situation of the runner must be selected as a pursuer to start the pursuing process. In the offered optimizer, the most suitable agent is a pursuer $\left({\vec{P}}_{b}\left(t\right)\;\right)$ that demonstrates the state of the runner $\left({\vec{X}}_{r}\left(t\right)\right)$ on the playing area.

#### 2.1.3 Exploitation and exploration (touch out and follow)

In the offered optimizer, the players have been sorted into two sets that includes; odd-numbered and even-numbered groups. Whenever a runner makes random movements in the playing area, two groups of pursuers demonstrate reactions with various attitudes. The tactic picked via a pursuer is totally correlated to a runner's state. Whenever a runner is located in front of one group, it is followed by a pursuer of the individuals of this group. Such an approach is addressed by a chase policy. Simultaneously, the other set of players remain motionless, and they address the unmoved policy. Based on the situation of a runner, two probabilities could happen.

− 1: Pursuers whose state is odd-numbered might follow the chase policy, and Pursuers whose place is even-numbered may utilize an unmoved policy.
− 2: The contrary of the first probability.

k is a factor that is established for the haphazard selection of every agent out of the above probabilities. Regarding the value k, a decision is made about the policy of two sets. The even-numbered agents and the odd-numbered ones will choose the unmoved and the chase policies if $k>\frac{1}{2}$. However, if $k\leq \frac{1}{2}$, the odd-numbered agents and even-numbered ones will opt the unmoved and the chase policies.

The renewing principle based on the chase policy could be mathematically stated in the following:

(3) $$\vec{u}\left(t+1\right)=\vec{u_{0}}\left(t\right)+\left({\vec{P}}_{b}\left(t\right)-{\vec{P}}_{b}{\left(t\right)}_{old}\right)\text{,}$$

(4) $${\vec{P}}_{f}\left(t+1\right)={\vec{P}}_{b}\left(t\right)-\left[g.\left[e.{\vec{P}}_{b}\left(t\right)-{\vec{P}}_{f}\left(t\right)\right]\right]+\vec{u}\left(t+1\right)\text{,}$$

here, ${\vec{P}}_{f}\left(t+1\right)$ specifies the renewed state of the pursuer who encounters the runner, ${\vec{P}}_{f}\left(t\right)$ signifies the pursuer situation vector who is opposed to the runner, ${\vec{P}}_{b}\left(t\right)$ illustrates the best pursuer situation vector that is also uttering the state of the runner, u(t) demonstrates the runner's comeback situation from the previous time instant, u(t+1) depicts the renewed comeback situation, *t* and t+1 indicate the present and subsequent chunks of time, g is a break randomizing operator that is as a scalar factor, and e is the endurance coefficient varying from 0 to 2 , illustrating the runner's grade of strength.

For renewing, the mathematical model based on unmoved policy can be expressed as the formula below:

(5) $${\vec{P}}_{o}\left(t+1\right)={\vec{P}}_{o}+g.\left[e.\;{\vec{P}}_{b}\left(t\right)-{\vec{P}}_{o}\left(t\right)\right]\text{,}$$

where, ${\vec{P}}_{o}\left(t\right)$ are the vectors of the pursuer situation settled opposite the runner.

g is an operator correlated to the chaser stamina factor (e). For its definition, the subsequent formula can be utilized:

(6) g=[(2.e.rnd)−e],

here, rnd represents a random value, varying from zero to one.

(7) $$e=\left[e_{T}-\delta_{e}.e_{T}\right]\text{,}$$

The runner's entire endurance is illustrated by $e_{T}$; however, it takes a value that is correlated to a fresh runner at its run commencement. $\delta_{e}\left(t\right)$ demonstrates the declination level of the runner's stamina that can be determined as:

(8) $$\delta_{e}\left(t\right)={\left(t/t_{R}\right)}^{2}$$

The present time and the runner's execution period are represented by t and $t_{R}$.

### 2.2 Stamina factor(e)

Pursuing and running is the principle of the Kho-Kho game. One vital physical factor, which impacts the performance of the runner, is the endurance operator. During the game, the runner becomes exhausted which causes the shrinkage of the stamina factor and influences the runner's place on the playground. When e=2, the level of endurance is great. However, the level of stamina is zero whenever e=0. 1<e<2 demonstrates high-mean endurance, and 0<e<1 illustrates the low-mean endurance. The runner's endurance starts decreasing during the run as much as the runner stops running. This declining rate is demonstrated by δ(e). During the game, there is always a particular space between the runner and the pursuer. When the mentioned space touches zero (its minimum value), the pursuit process is prosperous [18]. This space varies throughout the game, and there is a correlation between it and physical features, namely decreased stamina, barriers, speed fluctuations, and fatigue. Therefore, a gap randomizing means (g) has been introduced that its value has been found to be based on the runner's stamina (e).

### 2.3 Global optimization description

By selecting the pursuers (agents) and a runner (the finest agent), the process of optimality begins using the offered Kho Kho procedure. In the suggested approach, the key aim defines pursuing the finest member and ultimately discovering the finest global result, being the ultimate criterion. If pursuing the finest member is the aim, an eye must be kept on the place variation of the finest agent. According to it, other members must be developed. In a proper optimizer, alterations in the main member have to reflect on other members efficiently. In the represented optimizer, two objects of member improvement exist. In both of them, agents develop themselves by assuming the finest member's motions (the pursuer).

The only dissimilarity is that in the first object, there is an extra speed improvement, whereby the runner settles opposite the pitch. The improvement of speed is the change in the situation of the finest agent following its previous place, while the initial improvement is the location changes of the finest agent following the other agents. Subsequently, knowing both changes aids the other agents for effective change toward improvement [19]. For the global solution achievement, there must be random variations in the distinct search sphere and these changes have to go down during the execution of the optimizer. The random and lessening features of motions are expressed by the space-changing (g) and stamina (e) factors.

Consequently, the optimizer never gets stuck in local optimum results, and the finest global results get obtained. In the suggested optimizer, the obtainable epoch number for exploration is far more in comparison with those for exploitation. The search converges toward the result when (|g|)<1. In contrast, if (|g|)≥1, the search moves away from the result. There is a key focus on the situation of (|g|)≥1, because a more suitable use of the solution space is implemented by the optimizer. In the offered optimizer, the ultimate major phase is the ultimate criterion. During this phase, every optimum result is announced as the global result. In the represented Kho-Kho approach, the ultimate criterion happens whenever an active and robust pursuer follows the runner as much as its endurance shrinks to 0, so the pursuer touches its competitor out. In fact, e=0 is an indicator of the ending criterion for the suggested Kho-Kho optimizer.

### 2.4 Optimizer validation

For the assessment of an optimizer's operation, many terms of the procedure could be utilized. In this paper, for authorization of the capacity of the offered Kho-Kho optimizer, 10 test functions have been utilized. In these functions, there have been two unimodal and multimodal functions. F1 to F5 have been five unimodal functions in which distinctive global optimum points exist. The capacity of exploitation in the offered optimizer could be analyzed based on these functions. F6 to F10 is also a set of multi-modal functions, containing 5 functions [20]. A single global optimal solution does exist for mentioned functions, in addition to abundant local minimal ones. As a result, for evaluation of the optimizer's exploration potential, these functions are utilized [21]. The name of functions, their mathematical modeling, and the bounds of the search sphere have been illustrated in Table 1.

**Table 1:** Test functions for the investigation endorsement.

Equation	Function	Range
$F_{1}=\sum_{i=1}^{n}x_{i}^{2}$	Sphere	−100 < x < 100
$F_{2}=\sum_{i=1}^{n}\left|x_{i}\right|+\prod_{i=1}^{n}\left|x_{i}\right|$	Schwefel 2.22	−10 < x < 10
$F_{3}=\sum_{i=1}^{n}{\left(\sum_{j=1}^{i}x_{j}\right)}^{2}$	Schwefel 1.2	[-100,100]
$F_{4}=\lfloor \left|x_{i}\right|,1\leq i\leq n\rfloor$	Schwefel 2.21	[-100,100]
$F_{5}=\sum_{i=1}^{n-1}\left|100{\left(x_{i+1}-x_{i}^{2}\right)}^{2}+{\left(x_{i}-1\right)}^{2}\right|$	Rosenbrock	[-30,30]
$F_{6}=-20\times \exp\left(-0.2\times \sqrt{\sum_{i=1}^{n}x_{i}^{2}}\right)-\exp\left(\left(\frac{1}{n}\right)\times \sqrt{\sum_{i=1}^{n}\cos\;\left(2\pi x_{i}\right)}\right)+20+e$	Ackley	[-32,32]
$F_{7}=\frac{1}{4000}\sum_{i=1}^{n}x_{i}^{2}-\prod_{i=1}^{n}\cos\left(\frac{x_{i}}{\sqrt{i}}\right)$	Griewank	[-600,600]
$F_{8}=\sum_{i=1}^{n}\left|x_{i}\;\sin\left(x_{i}\right)+\frac{x_{i}}{10}\right|$	Alphine 1	[-10,10]
$F_{9}=\prod_{i=1}^{n}\left|\sin\left(x_{i}\right)\sqrt{x_{i}}\right|$	Alphine 2	[-10,10]
$F_{10}=\sum_{i=1}^{n}{\left(x_{i}-1\right)}^{2}+\sum_{i=1}^{n}x_{i}x_{i-1}$	Trid	[-100,100]

The original Kho-Kho optimizer [22], WCO (World Cup Optimizer) [23], FA (Firefly Algorithm) [24], and EHO (Elephant Herding Optimizer) [25] have been published as metaheuristic optimizers whose results are utilized for being compared with the offered method's outcome in this part. Table 2 depicts the variable set for the procedures.

**Table 2:** Parameter settings for the studied procedures.

Approach	Variable	Amount
WCO [23]	ac	0.2
	Playoff	0.03
EHO [25]	R	850
	β	0.05
	nClan	3
	γ	0.03
	α	0.35
FA [24]	γ	1
	β	0.7
	α	0.3

The procedures of optimality might not generate a globally optimal outcome because of the random swarm initial amount. They can, however, calculate a suboptimal outcome adjacent to an optimal result. Accordingly, 35 modellings of every function have been executed. Due to this subject, the determination of average (AVG) and standard deviation becomes (STD) simpler [26]. STD does help the process of variance assessment in the results, whereas AVG does provide the mean results for the 35 modellings. Having smaller average amounts is desirable, because the purpose is the function minimizing rather than resolving these functions. What is more, each term's disparity ought to be moderate as it is achievable. For running the entire 35 scenarios, 200 iterations are utilized. The results of the offered Kho-Kho optimizer and other procedures' outcomes are represented in Table 3.

**Table 3:** Modeling outcomes of the Self-adapted Kho-Kho optimizer and other approaches.

Benchmark		SAKhoKho	Kho-Kho [22]	EHO [25]	FA [24]	WCO [23]
f1	AVG	0.00	5.42	7.16	6.23	5.02
	STD	0.00	3.84	5.10	4.02	3.35
f2	AVG	3.97	6.15	6.34	7.22	5.71
	STD	3.04	5.56	5.11	6.18	5.09
f3	AVG	0.00	5.4E-12	7.12E-11	4.5E-10	5.64E-10
	STD	0.00	0.009	7.01E-6	4.5E-8	5.76E-5
f4	AVG	0.00	0.00	9.12E-8	6.1E-9	8.59E-9
	STD	0.009	0.04	8.64E-10	5.65E-10	7.71E-10
F5	AVG	0.00	4.12	8.24	6.43	5.16
	STD	0.00	5.97	8.52	7.71	6.61
F6	AVG	0.03	8.05	9.81	9.32	7.72
	STD	0.00	4.96	5.71	5.20	5.01
F7	AVG	0.00	8.01	8.72	7.53	8.16
	STD	0.713	2.85	4.18	4.52	4.01
F8	AVG	0.009	0.07	3.78	2.62	3.02
	STD	0.076	1.1	5.72	4.51	4.23
F9	AVG	0.00	0.00	2.82	1.62	2.14
	STD	0.00	0.00	7.42	6.61	7.11
F10	AVG	0.00	0.35	6,92	5.81	5.45
	STD	0.00	0.72	3.24	2.04	2.32

Regarding Table 3, the suggested SAKho-Kho gives the finest results for the group of uni-modal functions (F1–F5). Compared to other approaches, the result of standard deviation has also a minimum value for F1–F5. Such a behavior illustrates the offered procedure's superior exploitation capacity. Additionally, the offered SaKho-kKho optimizer gives the finest results for the set of multimodal functions (F6–F10), which depicts the strong exploration capacity of the offered procedure.

## 3 Rigdelet neural network (RNN)

The RNN is a unique ANN built on the Ridgelet transform, which differs from traditional classification neural networks in that it makes use of a non-linear excitation function [27]. Excitation functions of neurons frequently represent the input or output properties of a neural network which is significant for how well neural networks function. A Ridgelet neural network model inspires the directionality displayed by neurons.

Ridgelet, a higher-dimensional extension of wavelet, outperforms the traditional neural network in terms of performance. The RNN boosts directional depiction in extra situations and scales to more accurately represent the directional sensitivity of neurons. An RNN may estimate a complete function with multiple variables on a slighter measure. Since Ridgelet converts are comparatively stable, it offers a more flexible configuration, improved robustness, fault tolerance, and quicker parallel processing velocity [28]. Considering $\Phi :R^{n}\to R$, following condition must be applied:

(9) $$K_{\varphi }=\int \frac{{\left|\hat{Ϝ}\left(\omega \right)\right|}^{2}}{{\left|\omega \right|}^{M}}d\omega <\infty$$

where, M describes the spatial magnitude of the load forecasting approach, and $\hat{Ϝ}$ defines the FT (Fourier Transform) of Ϝ, while,

(10) $${\;Ϝ}_{\beta }\left(Z\left(t\right)\right)=a^{-\frac{1}{2}}\;Ϝ\left(\frac{<U,Z\left(t\right)>-b}{a}\right)$$

where, β determines the term Ridgelet in space Β, therefore:

(11) Β=(β=(a,b,U),a,b∈R,a>0,U∈USM,||U||=1),

where, USM determines an M-dimensional unit sphere space, U describes the Rigdelet positioning,

a describes the scale of the Ridgelet, and b defines the Ridgelet position.

The Ridge iterative function provides faster operation than Fourier and wavelet transform which makes it so beneficial to be used for estimating different functions. The estimated output formula can be generated by the Rigdelet functions. When important variable and the function $y|y=f\left(x\right):R^{n}\to R^{m}$ is split into m projection $R^{n}\to R$, the output is obtained as follows:

(12) $$\begin{array}{l} WP\left(t\right)=\sum_{i=1}^{K}w_{i}\times \varphi \left(\frac{<U_{i},Z\left(t\right)>-b_{i}}{a_{i}}\right)\\ Z,U_{i}\in R^{n};\;\left\|U_{i}\right\|=1 \end{array}$$

here, K determines the concealed layers' quantity.

**Fig. 1:**
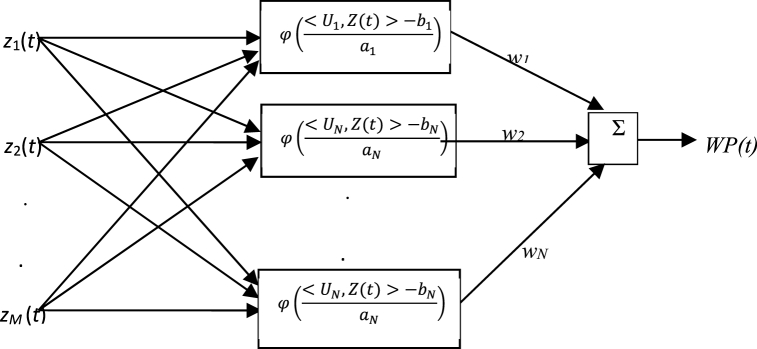
Rigdelet neural network's arrangement.

Fig. (1) demonstrates the Rigdelet neural network's arrangement.

Concerning Fig. (1), the lined amalgamation of the Ridgelets and weights ($w_{i}$) have been employed for increasing the efficacy of forecasting. The first step in the combined prediction arrangement is defining the RNN's variables. The training samples are first taken into consideration in the following way:

(13) $$X=\left[w_{1},w_{2},\ldots ,w_{K},a_{1},a_{2},\ldots ,a_{K},b_{1},b_{2},\ldots ,b_{K},U_{1},U_{2},\ldots ,U_{K}\right]$$

where, W, a, and b signify coefficients of Rigdelet.

The final parameter of the Rigdelet function includes the direction. This term can be formulated as follows:

(14) $$U_{1}=\left[u_{11},u_{12},\ldots ,u_{1m}\right],U_{2}=\left[u_{21},u_{22},\ldots ,u_{2m}\right],\ldots ,U_{K}=\left[u_{K1},u_{K2},\ldots ,u_{Km}\right]$$

To provide proper training of the Rigdelet neural network by considering their characterizations, the mentioned decision parameters ought to be optimally chosen. The idea here is to optimally select these decision variables such that the value of MSE (Mean Square Deviation) between the model's outcome and the preferred output has been minimized.

(15) $$MSE=\frac{1}{T}\sum_{j=1}^{L}\sum_{i=1}^{M}e_{ij}^{2}\left(t\right)$$

(16) $$e_{ij}\left(t\right)=d_{ij}\left(t\right)-y_{ii}\left(t\right)$$

where, $d_{ij}\left(t\right)$ and $y_{ij}\left(t\right)$ represent the network's anticipated outcome and the model's result that is achieved via the formula below:

(17) $$y_{ij}\left(t\right)=f\left(\sum w_{li}z_{ip}+w_{l0}\right)$$

where, l=1,2,…,m.

The purpose of this technique is to minimize Eq. (16), which is the error function used to evaluate the performance of the Ridgelet Neural Network (RNN) model. The decision variables in this equation are W, a, and b, which are the weights, scaling factor, and bias of the RNN, respectively. The self-adapted Kho-Kho optimizer is proposed as a way to provide the best decision variables for the RNN model and to address the limitations of earlier training methods. The optimizer is designed to efficiently search for optimal values of the decision variables that minimize the error function [27]. The self-adapted Kho-Kho optimizer is a powerful tool that enables the RNN model to learn from the data and improve its accuracy over time.

The contribution of the proposed method is significant, as it offers a more accurate and efficient way to train the RNN model for load forecasting. By using the self-adapted Kho-Kho optimizer, the proposed method can provide more accurate predictions of energy demand, which can benefit power system operators in making informed decisions about energy generation and distribution.

## 4 Wavelet transforms

One of the significant mathematical operations used in a variety of scientific disciplines is the wavelet transform. Wavelet transform's primary goal is to improve upon Fourier transform's flaws and restrictions. This transformation, as opposed to the Fourier transform, can be applied to dynamic systems and non-stationary signals. After the stage of decomposition, the number of samples decreases. Discrete wavelet transformation is applied to the signal in the form of a low pass in each pair of high-pass filters. A signal's output consists of two details followed by a general approximation signal. The analysis in the action stage is continued by analyzing the broad approximation to each signal. For applying the mother wavelet, φ(t), the following condition must be satisfied:

(18) $$\int_{-\infty }^{\infty }\emptyset \left(t\right)dt=0$$

Two Dealation and Translation operators examine the mother wavelet's functions, which are denoted by the following equation, when the investigated signal changes in size or location.

(19) $$\begin{array}{l} \emptyset_{b,a}\left(t\right)=\frac{1}{\sqrt{\left|a\right|}}\emptyset \left[\frac{t‐b}{a}\right],\\ a,b\in R,a\neq 0 \end{array}$$

where, b stands for the transfer (time) parameter, a illustrates the scale (frequency) parameter, and R signifies the restriction of the real values.

Wavelet transforms come in two varieties: continuous and discrete in time. Different types of wavelet transforms vary from one another, depending on how scale and transfer are accomplished. The following is the continuous wavelet transform of a time series with the function:

(20) $$\text{CWT}\left(a,b\right)=W_{\emptyset }f\left(a,b\right)={\left|a\right|}^{-\frac{1}{2}}\int_{-\infty }^{\infty }f\left(t\right)\bar{\emptyset }\left(\frac{t-b}{a}\right)dt$$

where, $\bar{\emptyset }$ is a function of ∅, which is expressed as follows:

(21) $$\text{CWT}\left(j,k\right)=W_{\emptyset }f\left(j,k\right)=a_{0}^{-\frac{j}{2}}\int_{-\infty }^{\infty }f\left(t\right)\bar{\Psi }\left(a_{0}^{-j}t-kb_{0}\right)dt$$

where, $a=a_{0}^{j}$, $b=kb_{0}a_{0}^{j}$, $a_{0}>1$, b∈R, and j∈Z.

The electricity demand time series in this research is originally perceived as a synthesis of distinct components with different scales and fluctuation levels. This research proposes that wavelet decomposition should be achieved in order to build a predictive RNN model for each component of the original series. All that is left is a rough (combined) series, which will be also modeled using an RNN. The RNN model and the wavelet transform are two names for this method. By combining the predictions for the components and the smoothed series, the prediction for the main series is produced.

**Fig. 2:**
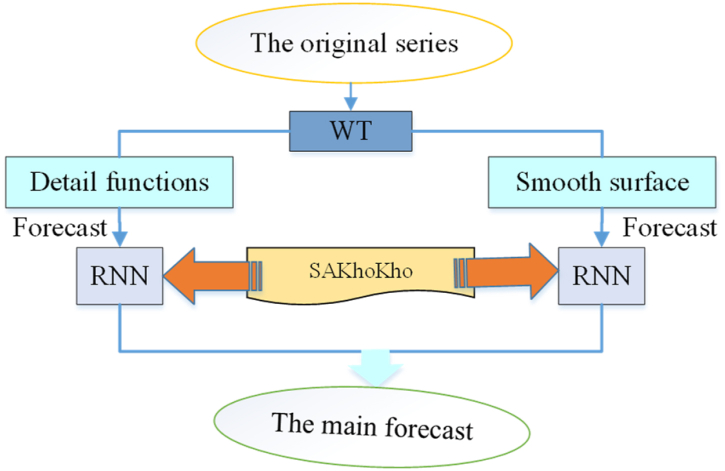
Model of the RNN/SAKhoKho/WT model.

The data is splatted into three phase levels using the Daubechies 5 wavelet (db5) to evaluate the efficacy of the analysis level. The details of levels one, two, and three are acquired for the smoothed level, which is modeled with a neural network. Taking the factors into account affecting the daily power demand, such as the days of the week, the average air temperature, and special holidays, the neural network's input layer's nine neurons have been included in all three levels. Fig. (2) depicts the RNN-wavelet transform model in RNN/SAKhoKho/WT form.

## 5 Simulation results and discussions

**Fig. 3:**
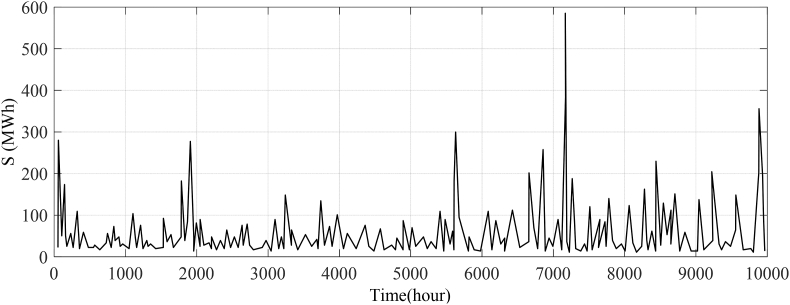
Preliminary settlement data for the areas of electricity market regulation.

As a result of a metaheuristic-based model and its stochastic nature, the outcomes may change each time it is used. With the parameter settings listed in Table 2, each experiment is run 15 times. In addition to the proposed SAKhoKho algorithm, some other cutting-edge approaches, including the Support vector machines with modeled annealing procedures [29], hybrid model [30], ARIMA model [31], MLP/PSO [32], CNN [33], and RNN/KhoKho/WT, were used and run on Matlab 2018b which was installed in an Intel core i5 computer with a 2.5 GHz processor and 4 GB memory. The preliminary settlement data for the Energy Market (PJM) regulatory area [34] used in this analysis and the capacity clearing price for each data is shown in Fig. (3).

The results presented in Fig. 3 show the preliminary settlement data for the PJM regulatory area used in the analysis as well as the capacity clearing price for each data. The data have been updated every two weeks based on the Intercontinental Exchange (ICE) figures. This indicates that the data used in the analysis is up-to-date and reflects current market conditions.

The fact that there are more than a dozen hubs and delivery points in North America where power supplies can be switched suggests that there is a complex and dynamic electricity market in the region. The use of ICE and EIA validation to cover eight main electricity hubs highlights the importance of accurate and reliable data in the analysis of electricity markets.

Generally, the results presented in Fig. (3) provide valuable information about the preliminary settlement data for the PJM regulatory area and the capacity clearing price for each data. The use of up-to-date data and validation from reliable sources, such as ICE and EIA, highlights the importance of accurate data in the analysis of electricity markets. These results are relevant to researchers and practitioners interested in analyzing electricity markets, as they provide insights into current market conditions and trends.

The two measurement indicators employed in the investigation have been considered to be the MAE (Average Absolute Deviation) and the RMSE (Root Mean Square Error). The mathematical formulas of the aforementioned assessment indicators are as follows:

(22) $$\text{RMSE}=\sqrt{\frac{\sum_{i=1}^{N}{\left(x_{i}-{\hat{x}}_{i}\right)}^{2}}{N}}$$

(23) $$\text{MAE}=\frac{1}{N}\sum_{i=1}^{N}\left|x_{i}-{\hat{x}}_{i}\right|$$

**Fig. 4:**
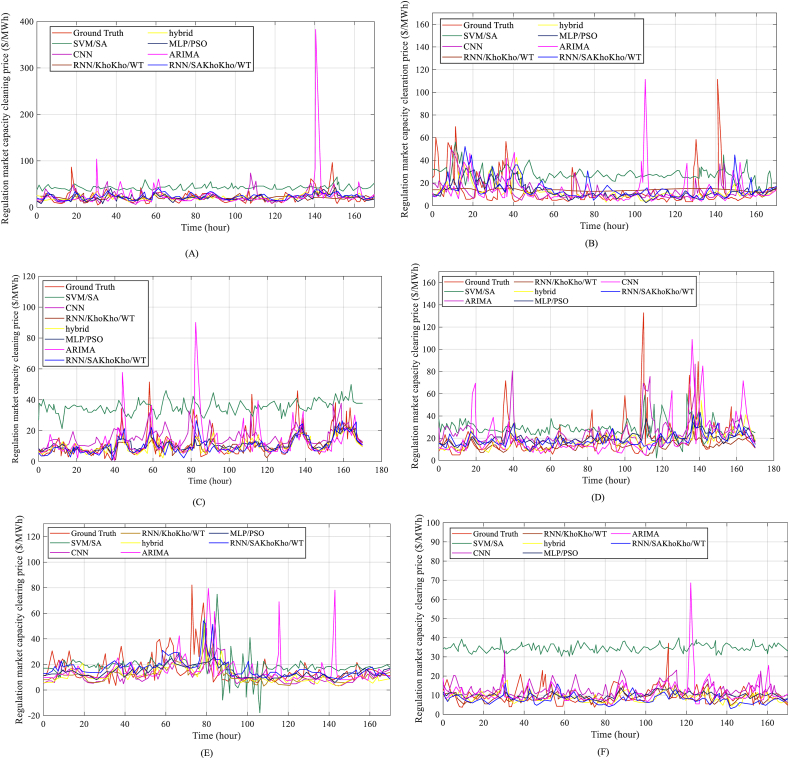
Outcomes of all prediction techniques' comparisons.

Six components from the database were selected to establish the model. The entire database is separated into 20% of test set and 80% of training set. Fig. (4) compares the outcomes of all prediction strategies.

From the results presented in Fig. (4), it is evident that the proposed RNN/SAKhoKho/WT model's efficiency in predicting energy prices is marginally lower than other methods. The model failed to achieve the power price and trend, resulting in extremely unreliable forecasting outputs with no matches to the real data. However, the hybrid and CNN models performed somewhat better than MLP/PSO in terms of determining the total trends in data, although the deviation was still significant. ARIMA produced more false maximum amounts. The paper's proposed strategy has the strongest experimental data support, indicating the efficacy and accuracy of the suggested approach for estimating electricity prices. The results presented in Table 4 and Table 5 provide further evidence of the accuracy of the proposed model, with low RMSE and MAE values. Overall, the proposed model appears to be an effective approach in energy price prediction, outperforming several other techniques.

**Table 4:** Comparing the outcomes of MAE of the modeling for the suggested RNN/SA KhoKho/WT and other analyzed methods.

No.	SVM/SA [29]	hybrid [30]	ARIMA [31]	MLP/PSO [32]	CNN [33]	RNN/KhoKho /WT	RNN/SA KhoKho/WT
1	5.9064	6.3570	6.1183	6.0153	20.8615	5.9351	5.1381
2	9.7235	10.7512	10.3415	9.9761	19.0478	10.2384	9.7410
3	7.6752	6.9180	6.1674	7.7452	16.6325	7.7143	7.3271
4	7.1062	5.5241	4.8822	7.3071	10.1861	6.5618	5.0768
5	8.3627	6.8365	6.2518	8.2017	20.2343	8.3021	6.5381
6	6.7678	5.1852	4.5578	6.6028	20.5513	5.7156	5.0425
7	13.0271	15.0041	11.9481	13.0183	39.7061	13.5017	12.8282
8	8.2561	11.0145	10.1568	8.2371	29.7021	9.5383	7.8254
9	11.1352	12.3201	11.6035	11.1054	41.2039	11.1512	11.0043
10	10.5510	6.2457	7.3227	10.7440	62.2385	10.6023	7.1558
Mean	8.75731	8.9254	8.13632	8.90323	28.04761	8.87192	7.7704

**Table 5:** The comparison outcomes of RMSE of the modeling for the offered RNN/SA KhoKho/WT and other analyzed methods.

No.	SVM/SA [29]	hybrid [30]	ARIMA [31]	MLP/PSO [32]	CNN [33]	RNN/KhoKho /WT	RNN/SA KhoKho/WT
1	11.2154	18.8513	11.7241	11.2461	21.2571	11.3121	10.0023
2	19.1342	20.1626	19.8046	19.1032	23.5642	19.1242	16.7472
3	12.9143	15.7315	12.0863	12.8415	20.5571	12.9018	12.0321
4	10.6752	12.7421	10.3057	10.7519	13.5802	10.9301	9.5518
5	18.7531	19.6352	17.2761	18.8361	28.6241	18.7463	17.7251
6	10.5421	12.2460	10.2151	10.2708	22.9023	10.6322	10.0747
7 b	39.5268	44.5721	40.3511	39.7082	64.8312	39.6338	39.3262
8	18.9153	40.5024	24.3135	18.8931	37.2512	19.0170	18.5228
9	23.9142	31.9561	24.5345	23.9423	45.6425	23.9024	23.6457
10	16.8025	18.9451	18.9415	16.8521	32.2182	16.8351	16.3275
Average	18.23165	23.5572	18.9835	18.2462	31.0347	18.30241	17.4132

The results show that the suggested RNN/SA KhoKho/WT model outperforms all the other analyzed methods in terms of MAE, with a mean MAE of 7.7704. The next best method is the hybrid method, with a mean MAE of 8.9254, followed by ARIMA (mean MAE of 8.13632) and MLP/PSO (mean MAE of 8.90323). The SVM/SA method has a mean MAE of 8.75731. The worst-performing methods are CNN and RNN/KhoKho/WT, with mean MAE values of 28.04761 and 8.87192, respectively. These results suggest that the suggested RNN/SA KhoKho/WT model is a promising method for Short-term Forecasting of Electric Load in Distribution Networks. The use of the SA algorithm and KhoKho strategy in the RNN model can effectively improve the accuracy of load forecasting. Furthermore, the results demonstrate that the hybrid method is also a competitive approach for load forecasting. In general, the results highlight the importance of selecting appropriate methods for load forecasting and the potential benefits of using advanced techniques like RNN and SA algorithms. Accurate load forecasting can enable utilities to better manage their resources, optimize their operations, and reduce their costs, ultimately leading to more reliable and affordable electricity for consumers.

The results show that the suggested RNN/SA KhoKho/WT model outperforms all the other analyzed methods in terms of RMSE, with an average RMSE of 17.4132. The next best method is the hybrid method, with an average RMSE of 23.5572, followed by ARIMA (average RMSE of 18.9835) and MLP/PSO (average RMSE of 18.2462). The SVM/SA method has an average RMSE of 18.23165. The worst-performing methods are CNN and RNN/KhoKho/WT, with average RMSE values of 31.0347 and 18.30241, respectively. These results suggest that the suggested RNN/SA KhoKho/WT model is a promising method for Short-term Forecasting of Electric Load in Distribution Networks. The use of the SA algorithm and KhoKho strategy in the RNN model can effectively improve the accuracy of load forecasting. Furthermore, the results demonstrate that the hybrid method is also a competitive approach for load forecasting. Generally, the results highlight the importance of selecting appropriate methods for load forecasting and the potential benefits of using advanced techniques, like RNN and SA algorithms. Accurate load forecasting can enable utilities to better manage their resources, optimize their operations, and reduce their costs, ultimately leading to more reliable and affordable electricity for consumers. However, it is also important to note that RMSE is not the only metric to evaluate the performance of a forecasting model. Other metrics, such as MAE, accuracy, precision, recall, and F1-score, should also be considered to gain a comprehensive understanding of the model's performance.

## 6 Discussions and remarks

The main results of the study demonstrate that the proposed method, which combines a Ridgelet Neural Network (RNN) with a wavelet transform and a self-adapted Kho-Kho optimizer, can significantly improve the accuracy of load forecasting. The results indicate that the proposed method outperforms traditional methods, such as ARIMA and LSTM, in terms of forecasting accuracy that has been measured by metrics, such as MAPE and Root Mean Squared Error (RMSE).

One insight that can be drawn from the results is that the use of the wavelet transforms for data preprocessing can significantly improve the accuracy of load forecasting. The wavelet transform allows for the extraction of relevant features from the data, which can improve the ability of the RNN model to capture the temporal dependencies in the data. This highlights the importance of data preprocessing in load forecasting and suggests that the wavelet transform is a useful tool for extracting relevant features from the data.

Another insight from these results is that the self-adapted Kho-Kho optimizer is a powerful tool for optimizing the RNN model and improving its accuracy. The optimizer efficiently searches for the optimal values of the decision variables, which can help overcome the limitations of traditional optimization algorithms. This suggests that the use of advanced optimization algorithms can significantly improve the accuracy of load forecasting and highlights the importance of optimizing the model's parameters.

These results demonstrate that the proposed method provides a significant improvement in the accuracy of load forecasting compared to traditional methods. The combination of the RNN architecture with the wavelet transform and self-adapted Kho-Kho optimizer allows the model to capture the temporal dependencies in the data and extract relevant features for accurate forecasting. This can benefit power system operators by providing more accurate predictions of energy demand, which can help optimize energy generation and distribution.

In addition, these results also highlight the importance of using up-to-date data and appropriate validation methods for accurate load forecasting. The use of the most recent data allows for more accurate predictions of energy demand, while appropriate validation methods, such as cross-validation, can help assess the accuracy and reliability of the model predictions.

In conclusion, the insights from the main results suggest that the proposed method provides a significant improvement in the accuracy of load forecasting and highlights the importance of data preprocessing, optimization algorithms, and validation methods in load forecasting. These insights can be valuable to researchers and practitioners interested in improving the accuracy of load forecasting and optimizing energy generation and distribution.

## 7 Complexity and the model's parameter uncertainty

The proposed model for load forecasting using the Ridgelet Neural Network (RNN) combined with a wavelet transform and self-adapted Kho-Kho optimization algorithm is relatively complex compared to traditional methods. The complexity of the model arises from the use of multiple layers of neurons in the RNN architecture, the wavelet transforms for data preprocessing, and the self-adapted Kho-Kho optimizer for model training.

The use of multiple layers of neurons in the RNN architecture allows the model to capture the temporal dependencies in the data, which are important for accurate load forecasting. However, this also increases the complexity of the model and can make it more difficult for training and optimization. The wavelet transform is used for data preprocessing to decompose the signals into different frequency bands. This allows for the extraction of relevant features from the data, but it also adds complexity to the model.

The self-adapted Kho-Kho optimizer is used for training model to optimize the decision variables of the RNN model. This is a powerful tool for improving the accuracy of the model, but it also adds complexity to the model. In terms of model's parameter uncertainty, the complexity of the proposed model can make it more difficult to estimate the model's parameters accurately. The use of the self-adapted Kho-Kho optimizer can help to address this issue by efficiently searching for the optimal values of the decision variables. However, there may still be some uncertainties in the estimated parameters due to the complexity of the model and the potential for overfitting the training data.

## 8 Conclusions

Short-term load prediction is crucial for the operating and design of energy systems, enabling the planning of unit entry and exit while considering production and network limitations. This importance increases in restructured power systems, where an accurate load forecast can lower electricity production costs and maximize usage. However, conventional techniques, such as regression analysis, time series models, and cause-and-effect models, may not provide precise results due to the nonlinear relationship between load and influencing factors. Intelligent systems, such as Neural Networks (NN), offer an effective approach to load forecasting. In this work, a Ridgelet NN was used for short-term load forecasting, combined with Wavelet Transform (WT) to improve accuracy by selecting suitable forecasting parameters. The Ridgelet Neural Network configuration was further improved using a self-adapted Kho-Kho optimization algorithm to enhance the precision of results. The present investigation model's training was founded on authentic Zone Preliminary Billing Data. To determine the superiority of the proposed model, it was subsequently compared with other approaches in the literature. The results indicated that the proposed approach outperformed all other scrutinized methods in both MAE and RMSE, with a mean MAE of 7.7704 and an average RMSE of 17.4132. The utilization of the SA algorithm and KhoKho strategy in the RNN model was a pivotal factor in enhancing its precision. The RNN/SA KhoKho/WT model's advantage was its capability to perceive the temporal dependencies in the data and optimize the model's weights to minimize the error function. The SA algorithm can efficiently search for the optimal weights, while the KhoKho strategy can further refine the optimization process by minimizing the search space. Moreover, the model's accuracy was evident in its performance compared to other methods. The hybrid method is the second-best approach, underscoring the potential benefits of amalgamating different forecasting methods to achieve better accuracy.

## CRediT authorship contribution statement

**Yaoying Wang:** Formal analysis, Data curation, Conceptualization. **Shudong Sun:** Software, Resources, Investigation, Formal analysis. **Gholamreza Fathi:** Writing – original draft, Supervision, Software, Resources, Conceptualization. **Mahdiyeh Eslami:** Writing – review & editing, Software, Resources, Methodology, Conceptualization.

## Declaration of competing interest

The authors declare that they have no known competing financial interests or personal relationships that could have appeared to influence the work reported in this paper.
